# Taurine Attenuates Calpain-2 Induction and a Series of Cell Damage via Suppression of NOX-Derived ROS in ARPE-19 Cells

**DOI:** 10.1155/2018/4596746

**Published:** 2018-07-29

**Authors:** Yuanyuan Zhang, Shu Ren, Yanting Gu, Jiahong Wang, Zheng Liu, Zhou Zhang

**Affiliations:** Department of Pharmacology, Shenyang Pharmaceutical University, Shenyang 110016, China

## Abstract

Nicotinamide adenine dinucleotide phosphate (NADPH) oxidases (NOXs) are key transmembrane proteins leading to reactive oxygen species (ROS) overproduction. However, the detailed roles of NOXs in retinal pigment epithelial (RPE) cell metabolic stress induced by Earle's balanced salt solution (EBSS) through starvation remain unclear. In this study, we investigated what roles NOXs play in regard to calpain activity, endoplasmic stress (ER), autophagy, and apoptosis during metabolic stress in ARPE-19 cells. We first found that EBSS induced an increase in NOX2, NOX4, p22phox, and NOX5 compared to NOX1. Secondly, suppression of NOXs resulted in reduced ER stress and autophagy, decreased ROS generation, and alleviated cell apoptosis. Thirdly, silencing of NOX4, NOX5, and p22phox resulted in reduced levels of cell damage. However, silencing of NOX1 was unaffected. Finally, taurine critically mediated NOXs in response to EBSS stress. In conclusion, this study demonstrated for the first time that NOX oxidases are the upstream regulators of calpain-2, ER stress, autophagy, and apoptosis. Furthermore, the protective effect of taurine is mediated by the reduction of NOX-derived ROS, leading to sequential suppression of calpain induction, ER stress, autophagy, and apoptosis.

## 1. Introduction

Reactive oxygen species (ROS) are signaling molecules that result in metabolic stress, changes in mitochondrial membrane permeability, DNA damage, and cell apoptosis [1–3]. ROS are generated by many cell types in the human body and are involved in the pathogenesis of various ocular diseases [4, 5], including glaucoma [6], age-related macular degeneration (AMD) [7], and retinopathy [8–10].

Nicotinamide adenine dinucleotide phosphate (NADPH) oxidase (NOX) is another key source of ROS besides mitochondria [11, 12]. NOX is the only enzyme group that produces ROS as its main function. Studies have shown that the NOX family is the inducer of ROS generation, ER stress, autophagy, and apoptosis [1, 13, 14]. There are different isoforms of NOXs in mammalian cells, containing NOX1–5 and DUOX1 and DUOX2 [15]. Of these homologues, we discovered that human retina expresses NOX1, NOX2, NOX4, and NOX5 [15–17]. Studies have shown that different homologues play different roles in retinal pathological processes. Some studies indicate that NOX1 is increased during eye disease and cardiac dysfunction [18, 19]. NOX2 is upregulated during ocular injury and diabetes [4, 16]. The expression level of NOX4 is increased during cardiomyocyte injury, diabetic retinopathy, and stroke [10, 13, 20]. The p22phox subunit is an essential part of the NOX compound. Except for NOX5 and DUOX1/2, p22phox is required for regulating NOX isoforms [3, 21]. Unlike other NOX homologues, NOX5 has the ability to bind and be activated by intracellular calcium directly and its function to produce ROS is regulated by intracellular calcium mobilization, influx, and phosphorylation [22, 23]. Calcium/calmodulin-dependent kinase II can activate NOX5 via direct phosphorylation [15]. Moreover, some studies show that crosstalk between ROS and calpain leads to the release of Ca^2+^ [24–27]. The nuclear translocation of calpain-2 can be activated by increased NOX-derived ROS. Furthermore, the ROS level and nuclear calpain-2 induction might be crucial pathogenic elements for apoptosis of cardiomyocyte [28].

There is growing evidence showing that NOXs are important sources of ROS during ER stress [29]. The increase of cellular stress and oxidative stress can lead to ER stress by activating the process of unfolded protein response and Ca^2+^ disturbances [30, 31]. Numerous studies have revealed that oxidative stress and ER stress are associated with neuronal cell death signaling after ischemia injury [32]. Oxidative stress plays an essential role in protective cell autophagy [33, 34]. ROS also leads to excessive autophagy and even apoptosis in cells [35]. It is known that apoptosis of ARPE-19 cells is the major cause of AMD-induced pathological changes [36]. In addition, a change in ROS balance is in charge of the execution of cell apoptosis [37, 38].

RPE cells are highly metabolically active cells that are located in the retina and play a vital role in maintaining normal visual function. Hence, RPE cells are vulnerable to oxidative stress [7, 39]. In addition, sunlight is one of the causes for ROS production that can damage RPE cells [40, 41]. The human cell line ARPE-19 has functional and structural characteristics similar to RPE cells. Therefore, we used an ARPE-19 cell line for our study.

In our previous study, we demonstrated that taurine inhibited starvation-triggered cell damage in ARPE-19 cells [24]. However, the detailed roles of NOX (one of the main ROS sources) in EBSS-induced cell injury remain unclear. We hypothesize that NOXs are the upstream regulators of calpains, ER stress, autophagy, and apoptosis. Furthermore, the protective effect of taurine is mediated by the reduction of NOX-derived ROS, leading to sequential suppression of calpain induction, ER stress, autophagy, and apoptosis. In this study, the potential roles of NOXs in EBSS-induced cell injury were studied in ARPE-19 cells.

## 2. Material and Methods

### 2.1. Cell Line and Cell Culture

ARPE-19 cells were procured from Shanghai GuanDao Biotech Co. Ltd. Cells were subcultured in Dulbecco's modified Eagle's medium (DMEM)/F-12 (Hyclone, Logan, UT, USA) containing 10% FBS (Gibco, Grand Island, NY, USA), penicillin (100 IU/mL), and streptomycin (100 *μ*g/mL). Cells were maintained at 37°C and 5% CO_2_.

### 2.2. Transfection Experiments

Human *NOX2*, *NOX4*, *p22phox*, and *NOX5* small interfering RNAs (siRNAs) were obtained from GenePharma (ShangHai, China). The siRNAs (two short sequences of siRNA specific for each one) were transfected into ARPE-19 cells using lipofectamine 2000 reagent (Invitrogen, Carlsbad, CA, USA) for 12 h. Next, cells were incubated for another 48 h in normal culture conditions. Protein expression was detected using Western blot, and the better siRNAs were chosen for subsequent experiments. The scrambled (nontargeting) siRNAs were used as the negative control.

### 2.3. Western Blotting

The protocol of the Western blot analysis is described in detail elsewhere [24]. Antibodies used and their dilutions were the following: NOX1 (1 : 500, Cambridge, MA, USA, Abcam, ab55831), NOX2/gp91phox (1 : 500, ABclonal Biotech, Wuhan, China, A1636), NOX4 (1 : 1000, Abcam, ab133303), p22phox (1 : 1000, Abcam, ab191512), and NOX5 (1 : 500, ABclonal, A7136). Other antibodies are described in detail elsewhere [24].

### 2.4. Flow Cytometry

Cellular ROS levels were monitored using the fluorescent probe (DCFH-DA). Cells were seeded in 6-well plates overnight and treated with EBSS for 24 h with or without inhibitors/siRNA/taurine pretreatment. Next, cells were loaded with DCFH-DA (10 *μ*M) in PBS for 30 min. Fluorescence was measured with a FACSCalibur flow cytometer (Becton, Dickinson and Company, San Jose, CA, USA). The protocol of the annexin V/PI staining is described in detail elsewhere [24]. The cytosolic-free calcium was detected using Fluo-3 AM. The protocol of the intracellular calcium detection is described in detail elsewhere [42].

### 2.5. Confocal Microscopy

Cells were cocultured with DCFH-DA (10 *μ*M, Beyotime Biotech, ShangHai, China) for 30 min. Nuclei were stained with Hoechst for 20 min. The protocol of image analysis is described in detail elsewhere [24].

### 2.6. Statistics

Data was expressed as the mean ± standard error of the mean (SEM). Statistics were analyzed by SPSS 21.0 statistics program (SPSS Inc., Chicago, IL, USA). One-way ANOVA and Dunnett's posttest were used to determine the statistical significance. A *p* value of less than 0.05 (*p* < 0.05) was considered significant.

## 3. Results

### 3.1. Effects of EBSS Treatment on NOX Expression and ROS Generation

**Figure 1:**
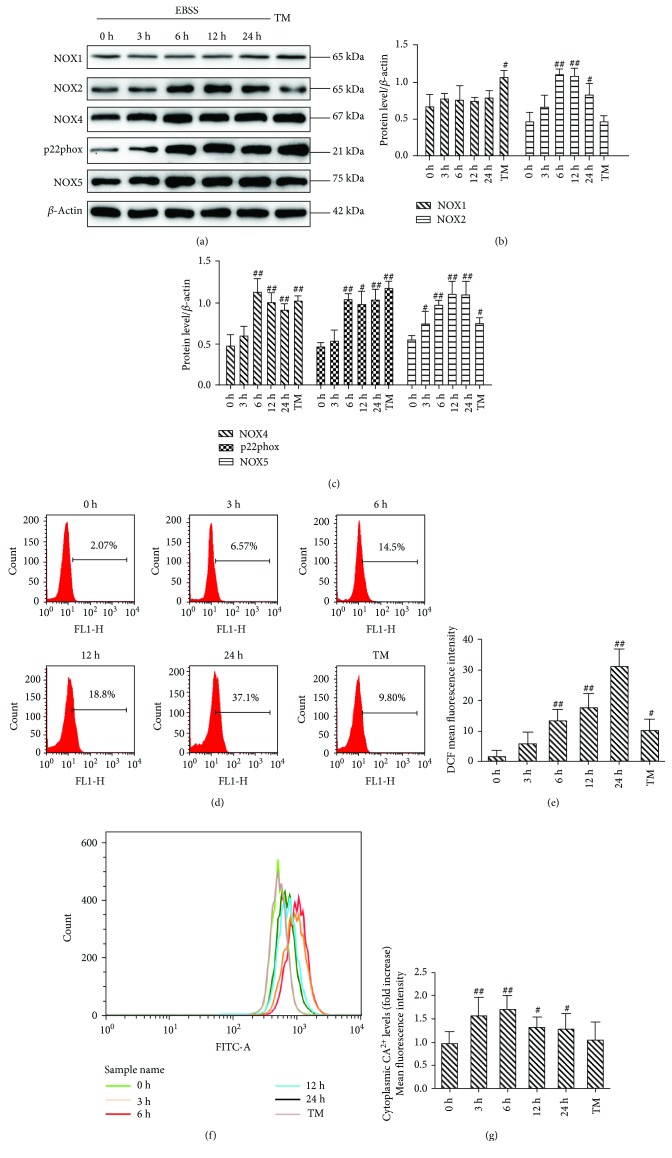
Earle's balanced salt solution (EBSS) increases NADPH oxidative activity and ROS generation in ARPE-19 cells in a time-dependent manner. (a–c) A Western blot analysis was carried out to detect the expression levels of NOXs. (d, e) Intracellular ROS was determined by flow cytometry. (f, g) Earle's balanced salt solution (EBSS) increases cytoplasmic calcium in ARPE-19 cells. The intracellular calcium was detected with Fluo-3 AM. TM stands for tunicamycin. The data are presented as the means ± SEM of three independent experiments. ^#^*p* < 0.05 and ^##^*p* < 0.01 compared to control (0 h).

To evaluate the role of EBSS treatment in the activities of NOXs and ROS generation, the cells were cultured in EBSS for 0, 3, 6, 12, and 24 hours (h) or with tunicamycin (50 *μ*M) as a positive control for 6 h. The expression of NOX proteins was examined by Western blot. The results show that the protein expression of NOX2, NOX4, p22phox, and NOX5 was dramatically increased from 6 h until 24 h after EBSS treatment. However, the expression of NOX1 was unchanged (Figures 1(a)–1(c)). The generation of ROS was markedly elevated between 6 h and 24 h after EBSS treatment (Figures 1(d) and 1(e)). Our results suggest that EBSS induces the activities of NOXs and ROS production. In addition, levels of NOXs were fully activated by 6 h posttreatment and subsequently this time point was chosen for our experiments. ER stress, autophagy, and apoptosis also could be induced at 6 and 12 h, respectively (data available online with this article; doi: 10.3390/ijms18102146, pages 4-5). We observed that the fluorescence intensity of the peaks shifted immediately after 3 h until 24 h post-EBSS treatment. Furthermore, EBSS induced the loss of calcium homeostasis in ARPE-19 cells (Figures 1(f) and 1(g)).

### 3.2. ROS Scavenging and NOX Suppression Attenuated EBSS-Induced Loss of Calcium Homeostasis and ER Stress

**Figure 2:**
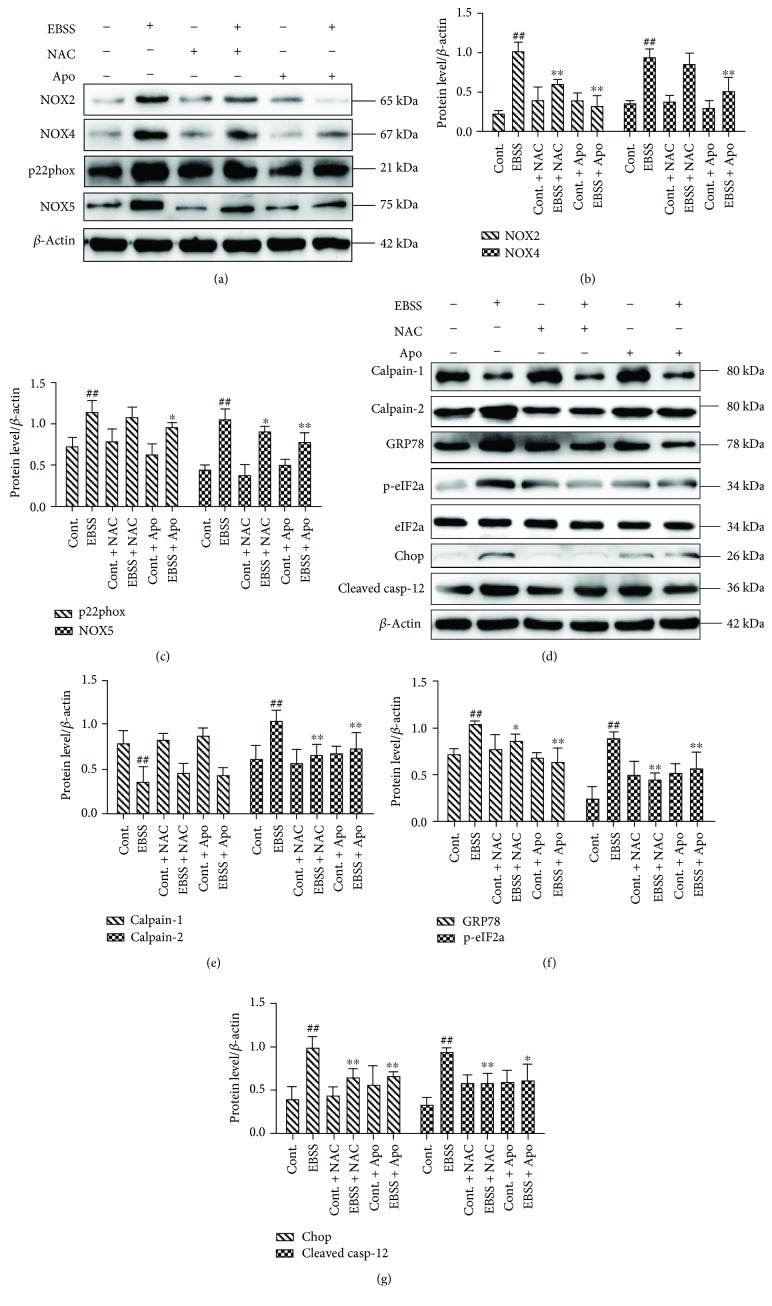
ROS scavenging and NOX suppression attenuate EBSS-induced loss of calcium homeostasis and ER stress in ARPE-19 cells. (a–c) The expression levels of NOX proteins were examined by Western blot. (d–g) The expression levels of calpains and the ER stress-related proteins were examined by Western blot. The data are presented as the means ± SEM of three independent experiments. ^##^*p* < 0.01 compared to the control group. ^∗^*p* < 0.05 and ^∗∗^*p* < 0.01 compared to the EBSS group.

To investigate the possible roles of NOXs and ROS in EBSS-induced loss of calcium homeostasis and ER stress in ARPE-19 cells, the cells were pretreated for 2 h with the ROS scavenger compound, N-acetyl-cysteine (NAC, 10 mM), or the NOX inhibitor, apocynin (Apo, 1 mM), and then cultured with EBSS (6 or 12 h). We found that pretreatment with NAC or Apo strongly and significantly reduced the expression of NOX2, NOX4, p22phox, and NOX5 (Figures 2(a)–2(c)), suggesting that the activity of NOXs and the accumulation of ROS were inhibited by the two inhibitors. To assess the relationship between NOXs, ER stress, and calpains in ARPE-19 cells, calpains and ER stress-related proteins were examined by Western blot. The results show that the expression of calpain-2 and ER stress-related proteins was decreased. However, induction of calpain-1 was unchanged (Figures 2(d)–2(g)). Our findings suggest that the induction of NOXs can promote loss of calcium homeostasis and ER stress. Furthermore, NOXs may be critical upstream regulators of calcium homeostasis and ER stress.

### 3.3. NOX Suppression and ROS Scavenging Attenuated EBSS-Induced Autophagy, Apoptosis, and ROS Generation

**Figure 3:**
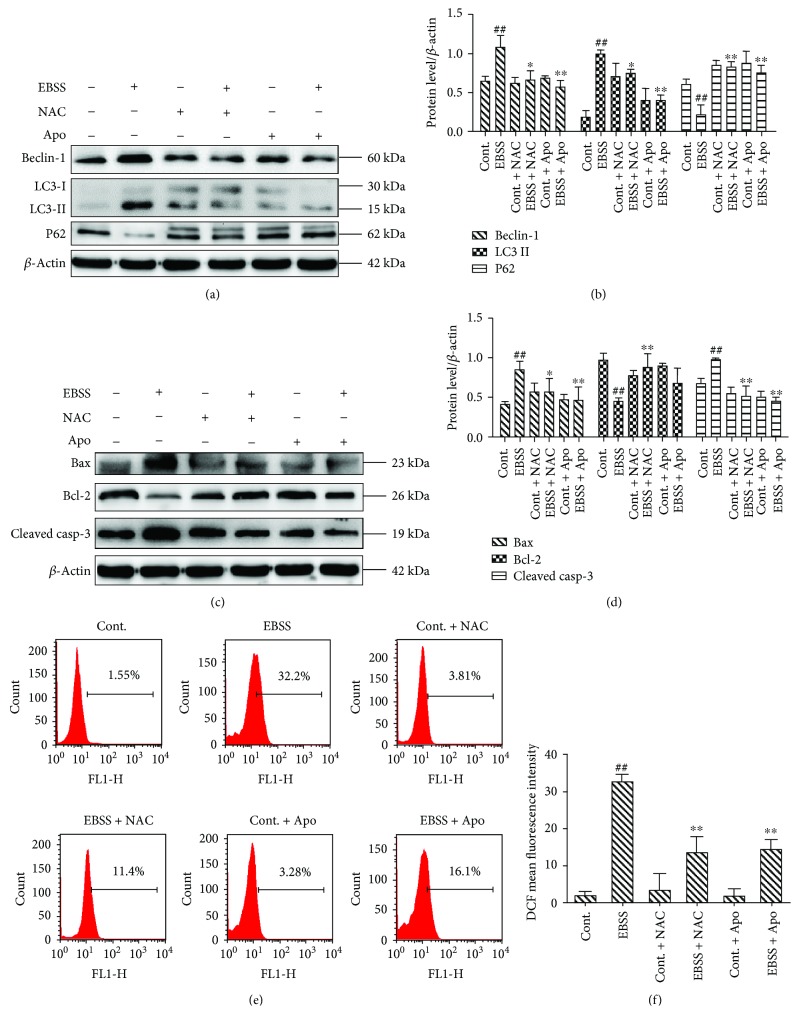
ROS scavenging and NOX suppression attenuate EBSS-induced autophagy, apoptosis, and ROS generation. (a–d) The expression of autophagy-related proteins and apoptosis-related proteins was detected by Western blot. (e, f) Intracellular ROS was evaluated by flow cytometry. The data are presented as the means ± SEM of three independent experiments. ^##^*p* < 0.01 compared to the control group. ^∗^*p* < 0.05 and ^∗∗^*p* < 0.01 compared to the EBSS group.

To further explore the possible roles of NOXs and ROS in EBSS-induced autophagy and apoptosis in ARPE-19 cells, we examined the expression of autophagy-related proteins and mitochondrial apoptotic proteins by Western blot. Our results suggest that both inhibitors, NAC and Apo, caused a significant decrease in the expression of beclin-1, LC3, Bax, and cleaved caspase-3. Meanwhile, levels of P62 and Bcl-2 were noticeably increased (Figures 3(a)–3(d)), indicating that the NOXs were related to the induction of autophagy and apoptosis. To evaluate the role of ROS, intracellular ROS was first evaluated by flow cytometry. The results show that pretreatment with NAC or Apo reverses the accumulation of ROS (Figures 3(e) and 3(f)). Our findings show that the suppression of NOX oxidases or ROS generation could decrease autophagy and apoptosis. Moreover, NOXs may be vital upstream regulators of ER stress, autophagy, and apoptosis.

### 3.4. NOX4, p22phox, and NOX5 Played Crucial Roles in EBSS-Induced Loss of Calcium Homeostasis and ER Stress

**Figure 4:**
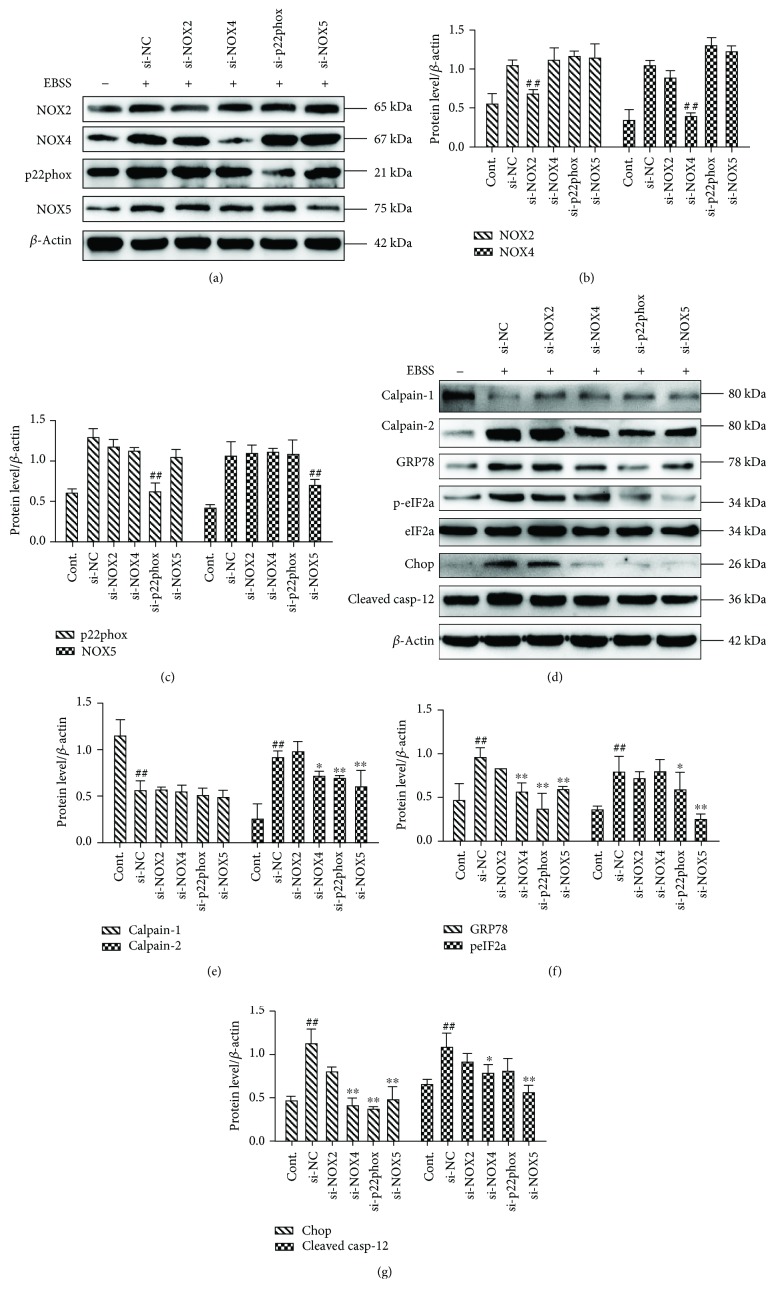
NOX4, p22phox, and NOX5 modulate loss of calcium homeostasis and ER stress in ARPE-19 cells. (a–c) Silencing of *NOX2/NOX4/p22phox/NOX5* decreases its protein level. (d–g) The activities of ER stress and calpain-2 were suppressed by siRNA knockdown of *NOX4*, *p22phox*, and *NOX5*. The data are presented as the means ± SEM of three independent experiments. ^##^*p* < 0.01 compared to the control group. ^∗^*p* < 0.05 and ^∗∗^*p* < 0.01 compared with the EBSS group.

To test the involvement of NOXs in EBSS-induced calpain production and ER stress, we used specific interference RNA (siRNA) species leading to a marked decrease of NOX protein levels (Figures 4(a)–4(c)). To demonstrate the effect of NOXs on ER stress and calpain production, calpains and the ER stress-related proteins were examined by Western blot. The results show that *NOX4*, *p22phox*, and *NOX5* silencing resulted in lower levels of calpain-2 and ER stress-related proteins. However, induction of calpain-1 was unaffected (Figures 4(d)–4(g)). Our findings indicate that silencing *NOX4*, *p22phox*, and *NOX5* can prevent calpain induction and ER stress. Furthermore, NOX4, p22phox, and NOX5 may be essential upstream regulators of calcium homeostasis and ER stress.

### 3.5. NOX4, p22phox, and NOX5 Play Key Roles in EBSS-Induced Autophagy, Apoptosis, and ROS Generation

**Figure 5:**
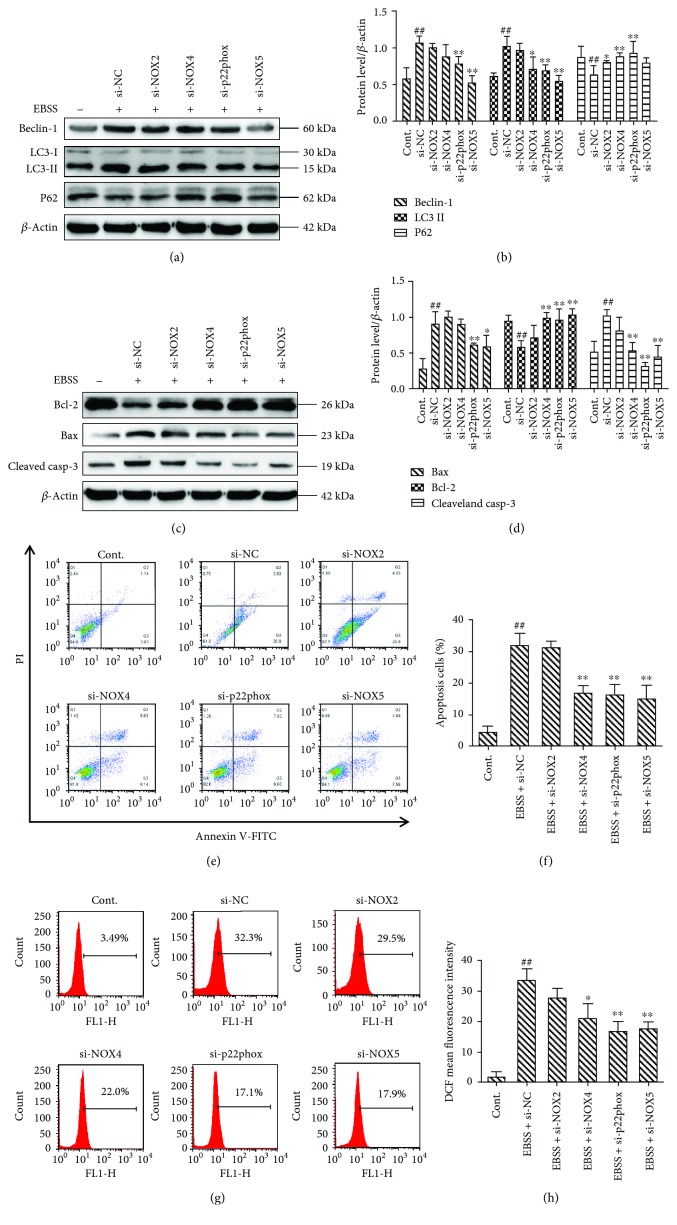
NOX4, p22phox, and NOX5 modulate autophagy and apoptosis in ARPE-19 cells. (a–d) NOX4, p22phox, or NOX5 silencing inhibits autophagy and apoptosis. (e, f) siRNA knockdown of *NOX4*, *p22phox*, or *NOX5* decreases cell apoptosis. (g, h) siRNA knockdown of *NOX4*, *p22phox*, or *NOX5* decreases ROS production. Intracellular ROS was evaluated by flow cytometry. The data are presented as the means ± SEM of three independent experiments. ^##^*p* < 0.01 compared to the control group. ^∗^*p* < 0.05 and ^∗∗^*p* < 0.01 compared to the EBSS group.

We next evaluated the effects of NOX2, NOX4, p22phox, and NOX5 on autophagy, apoptosis, and ROS production. The autophagy-related proteins and apoptosis-related proteins were examined by Western blot. The results show that *NOX4*, *p22phox*, and *NOX5* silencing downregulated the expression of beclin-1, LC3, Bax, and cleaved caspase-3. Meanwhile, the levels of P62 and Bcl-2 were increased (Figures 5(a)–5(d)). Moreover, the cell apoptosis rate was evaluated by flow cytometry, and our data show that apoptosis is inhibited with the reduction of NOX4, p22phox, and NOX5 expression (Figures 5(e) and 5(f)). The main sources of ROS are NOX enzymes, and our data suggest that the downregulation of NOX4, p22phox, and NOX5 is associated with significantly reduced production of ROS (Figures 5(g) and 5(h)). Our findings indicate that silencing of *NOX4*, *p22phox*, and *NOX5* can reduce autophagy, apoptosis, and ROS production. Furthermore, NOX4, p22phox, and NOX5 may be essential upstream regulators of autophagy and apoptosis.

### 3.6. Effects of Taurine on NOX Expression and ROS Generation Induced by EBSS

**Figure 6:**
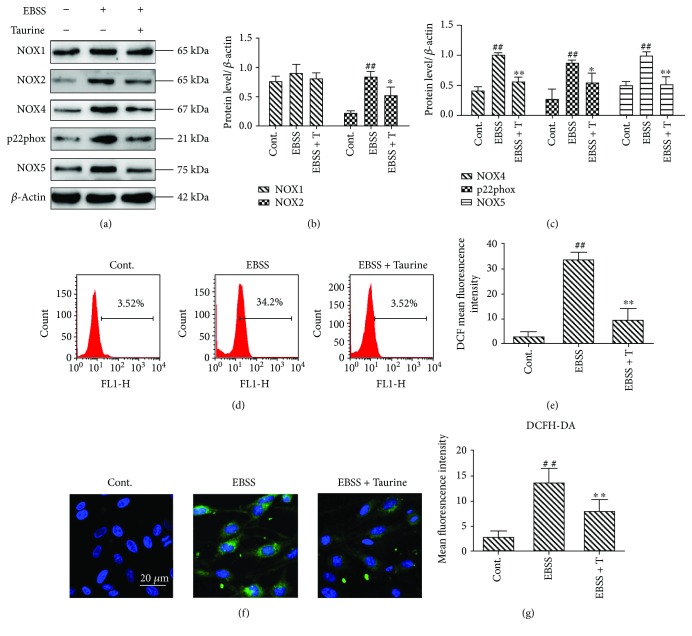
Effects of taurine on NOX expression and ROS generation induced by EBSS. (a–c) Western blot analysis was carried out to determine the expression of NOX proteins in ARPE-19 cells. (d, e) Intracellular ROS was evaluated by flow cytometry. (f, g) Confocal images of ROS labelled with DCFH-DA (green) and nuclear stained with DAPI (blue). Scale bar = 20 *μ*m. The data are presented as the means ± SEM of three to five independent experiments. ^##^*p* < 0.01 compared to the control group. ^∗^*p* < 0.05 and ^∗∗^*p* < 0.01 compared to the EBSS group. T: taurine.

We also investigated the effects of taurine (30 mM) on NOXs and ROS. Our results suggest that the expression of NOX2, NOX4, p22phox, and NOX5 in the EBSS group was increased compared with the control group. The level of proteins was decreased after intervention with taurine. The expression of NOX1 levels was not influenced in models (EBSS treatment) and taurine + EBSS groups (Figures 6(a)–6(c)). Oxidative stress was evaluated by detecting ROS with flow cytometry. Intracellular ROS was decreased by taurine treatment (Figures 6(d) and 6(e)). Moreover, ROS accumulation was also determined with immunofluorescence. High fluorescence intensity was presented in the EBSS group. But it was weak in the control and EBSS + taurine groups (Figures 6(f) and 6(g)). These data suggest that taurine inhibited the activities of NOX enzymes and the accumulation of ROS. Therefore, taurine attenuates ER stress, autophagy, and apoptosis in ARPE-19 cells via suppression of the NADPH oxidase-derived reactive oxygen species-mediated calpain induction pathway (data available online with this article; doi:10.3390/ijms18102146, pages 8-9).

## 4. Discussion

**Figure 7:**
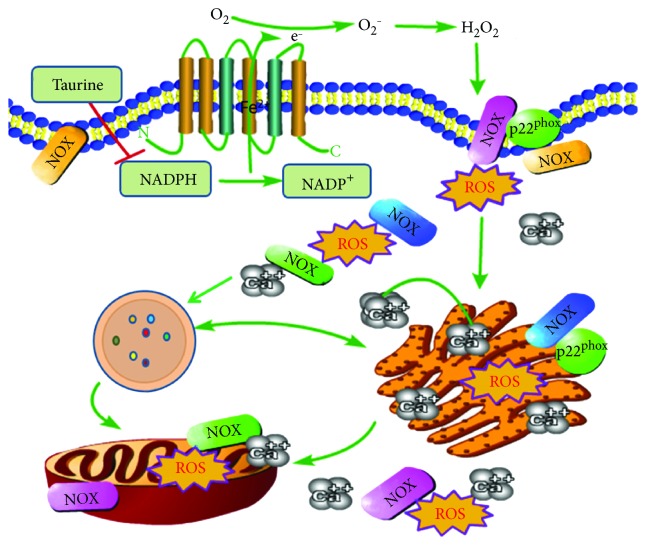
Schematic diagram of the effects of taurine in ARPE-19 cells. The protective effect of taurine is regulated by the reduction of intracellular ROS from NOXs, leading, in turn, to sequential suppression of calpain induction, ER stress, autophagy, and apoptosis. NADPH oxidases (NOXs) are transmembrane proteins that are localized either in intracellular granules and vesicles or on the cell surface membranes.

Our study demonstrates the involvement of NOX-derived ROS in EBSS-induced RPE metabolic disorders. NOXs are upstream regulators of calpain-2, ER stress, autophagy, and apoptosis. Furthermore, NOXs are involved in calpain-2, ER stress, autophagy, and apoptosis regulation by NOX4, p22phox, and NOX5 but not by NOX1 or NOX2. Taurine alleviated cell injuries through regulation of NOX levels (Figure 7).

During metabolic stress (starvation), the balance of ROS level changes. NOXs are activated and result in excessive production of ROS (termed oxidative stress) (Figure 1). Furthermore, an excessive level of ROS interferes with lipids and proteins leading to functional and structural changes of target molecules [43, 44]. NOX1/NADPH oxidase plays a key role in endotoxin-induced cardiomyocyte apoptosis [19]. NOX1 does not influence vascular endothelial growth factor (VEGF) activation in HG-induced cell injuries [16]. NOX2 and NOX4 play physiological roles in homocysteine-induced endothelial cell apoptosis [45]. NOX5 and p22phox are involved in human monocyte differentiation into dendritic cells [3]. We demonstrate that NOX4 and p22phox play key roles in ARPE-19 cell injuries (Figure 1). NOX5 is expressed in cells of the cardiovascular system and in the retina [46]. However, NOX5 is absent in rodents and this may explain the lack of data on its expression and function in the retina in this model. Previous research indicated that NOX5 was predominantly expressed in RPE cells [15]. Our results show that NOX5 is a key factor of EBSS-induced cell damage (Figure 1).

The interaction of ROS and calcium signaling can be considered bidirectional; ROS can regulate cellular calcium signaling, while calcium signaling is crucial for ROS generation [25, 47, 48]. Increasing evidence suggests that this crosstalk plays a key role in many pathological conditions, including colorectal cancer, cardiomyocyte apoptosis, neuronal damage, and also renal cell injury [28, 49–51]. We revealed that suppression of NOX-mediated ROS production through the use of the NOX inhibitor (apocynin) or ROS scavenger (NAC) inhibited calpain-2 induction (Figure 2). During cellular stress, NOX-mediated ROS generation was observed. ROS levels correlate with cell survival. The decrease in ROS production also reduces the expression of NOX, which forms a feed-forward loop [52–55]. NOX is activated due to its notable ability to produce ROS. The ER is a major site of calcium storage [25]. The release of Ca^2+^ also leads to ROS accumulation [56, 57]. Overproduction of ROS activated ER-based calcium channels by triggering signaling molecules upstream of calpain-2 [58–60]. Induction of calpain-2 further mediates the subsequent ER-mitochondrial crosstalk [1, 28, 50]. ROS not only acts as an activator but can also be regulated by autophagy through macroautophagosomes [34, 61]. Bcl-2 is an integral membrane protein, and it can form homodimers to protect cells from apoptosis. In contrast, Bax is a proapoptotic gene and can cause mitochondria to release cytochrome C. Cytochrome C leads to caspase 3 activation which is an important mitochondrial apoptotic marker [62, 63]. We investigated the ROS-calcium crosstalk in the ER, autophagosome, and their influence on apoptosis in the mitochondria. Our results indicate that suppression of NOX oxidases or ROS generation could decrease calpain-2 induction, ER stress, autophagy, and apoptosis (Figures 2 and 3).

To elucidate the functional significance of NOXs, we examined ROS generation, ER stress, autophagy, and apoptosis in which NOX2/NOX4/p22phox/NOX5 was knocked down with siRNA. Although NOX4/p22phox/NOX5 was not completely knocked down, induction of NADPH oxidase was inhibited. The exact reasons for this are complex and not well-understood but relate to the fact that NOX4/p22phox/NOX5 downregulation influences other NADPH oxidase subunits that may have an impact on the induction of oxidases [45, 64]. It is also possible that NOX4/p22phox/NOX5 might interact with other NOX isoforms, which, in the context of NOX4/p22phox/NOX5 downregulation, inhibits NOX-associated NOX induction. Such considerations require further examinations. In NOX4/p22phox/NOX5-downregulated cells, but not NOX2-downregulated cells, ROS generation, ER stress, autophagy, and apoptosis were significantly reduced, indicating the importance of NOX4/p22phox/NOX5 in redox signaling by EBSS. Moreover, these processes are Ca^2+^ sensitive, because calpain-2 induction was also inhibited. Hence, NOX influence generation of ROS also regulates ER stress, autophagy, and apoptosis. We showed that NOX4, p22phox, and NOX5 are critical for damage and cell death of ARPE-19 cells. In addition, we found for the first time that NOX5 induction is required for calpain-2 induction, ER stress, autophagy, and apoptosis in ARPE-19 cells (Figures 4 and 5).

The antioxidation of taurine may prevent disease progression [65]. The antioxidant properties are limited by the sulfonic group through neutralizing ROS production [41, 66]. In a previous study, we demonstrated that taurine inhibited starvation-triggered cell damage. In our study, our results suggest that taurine inhibited the activation of NOXs and ROS generation (Figure 6).

In conclusion, our data demonstrate that ROS generated via the NADPH oxidase system is the major contributing factor in RPE dysfunction induced by EBSS. NOX silencing or suppression partly protects cells from cell damage. NOX4, p22phox, and NOX5 play key roles in the production of ROS, ER stress, autophagy, and apoptosis. Our observations support the hypothesis that oxidative stress is the main causative for cell injury, and taurine attenuates ER stress, autophagy, and apoptosis in ARPE-19 cells via a suppression of the NADPH oxidase-derived reactive oxygen species-mediated calpain induction pathway. Our data provides novel insights into ocular diseases and mechanisms of taurine.

## Data Availability

Previously reported research data were used to support this study and are available at doi: 10.3390/ijms18102146. These prior studies (and datasets) are cited at relevant places within the text as references. The data used to support the findings of this study are available from the corresponding author, Zhou Zhang, upon reasonable request.
